# Psychometric properties of the Brazilian version of the lived experience component of the Spiritual Health And Life-Orientation Measure (SHALOM)

**DOI:** 10.1186/s41155-018-0083-2

**Published:** 2018-01-25

**Authors:** Sandra Adriana Neves Nunes, Helder Miguel Fernandes, John Wayne Fisher, Marcos Gimenes Fernandes

**Affiliations:** 1 Universidade Federal do Sul da Bahia, Rua Itabuna, s/n, Rod. Ilhéus-Vitória da Conquista, Km 39, Br 415, Ferradas, Itabuna, BA 45613-204 Brazil; 2 Research Centre in Sports Sciences, Health Sciences and Human Development, University of Trás-os-Montes and Alto Douro, Campus Desportivo, 5001-801 Vila Real, Portugal; 3 University of Melbourne, Grattan St, Parkville, Melbourne, Victoria 3010 Australia; 4 Federation University Australia, University Drive, Mt Helen VIC, Ballarat, Victoria 3350 Australia; 5 Universidade Estadual de Santa Cruz, Campus Soane Nazaré de Andrade - Rod. Jorge Amado, km 16 - Salobrinho, Ilhéus, BA 45662-900 Brazil

**Keywords:** Cross-cultural adaptation, Psychometric properties, Spiritual health, Brazilian population

## Abstract

This study had the following aims: (i) to translate the Spiritual Health and Life-Orientation Measure (SHALOM) into Brazilian Portuguese and adapt it to ensure the semantic/conceptual equivalence and content validity of the Brazilian version and (ii) to analyse the psychometric properties—reliability, convergent validity, discriminant validity and factorial validity—of the lived experience component, also called the Spiritual Well-Being Questionnaire (SWBQ), in a calibration sample and in a validation sample of Brazilian adults. The calibration sample comprised 436 subjects, 159 men and 277 women, aged between 18 and 79 years (mean age = 32.20 years; SD = 11.46); the validation study sample comprised 388 subjects, 253 women and 135 men, aged between 18 and 59 years (mean age = 30.59 years; SD = 9.44). All subjects completed a sociodemographic questionnaire, the Brazilian SWBQ and the Psychological Well-being Scale (PWBS). The results provide evidence of the reliability and factorial validity of an oblique four-factor model of a reduced 17-item version but revealed some problems with the convergent validity of the communal and personal factors (average variance extracted < .50). Nonetheless, these results provide evidence that the Brazilian version of the lived experience component of SHALOM (or SWBQ_b_) has good psychometric properties and is a valid method of evaluating the spiritual health of Brazilian adults. Further research is required to establish the convergent and discriminant validity of this reduced version.

## Background

For a long time, phenomenon of spirituality was neglected by the academic world as it was considered too subjective, hard to operationalise and to measure (Moberg, 2008), but over the past 30 years, there has been a notable increase in publications about spirituality and health (Weaver, Pargament, Flannelly, & Oppenheimer, 2006), due to advances in the operationalisation and measurement of spirituality as a construct (Moberg, 2008).

Spirituality and health are related to each other in that spirituality can be a source of support for people when they experience stress resulting from chronic disease or when they perceive their life is at risk (Smith, McCullough, & Poll, 2003; Stefanek, McDonald, & Hess, 2005; Tuck, McCain, & Elswick, 2001). Spiritual disposition seems to be associated with mental health (Koenig, McCullough, & Larson, 2001; Sawatzky, Ratner, & Chiu, 2005) as it has been shown to be a predictor of happiness, psychological well-being and lower stress (Rowold, 2011).

The concept of spiritual well-being or spiritual health (SH) emerged from research investigating the contribution of spirituality to human health (Fisher, Francis, & Johnson, 2000; Moberg, 2008). According to Fisher (2010), spiritual well-being can be understood as good spiritual health and an indicator of spiritual quality of life, an aspect of human health that goes beyond physical, psychological and social dimensions. In 1975, the National Interfaith Coalition on Ageing (NICA) presented a broad, consensual definition of spirituality, proposing that “spiritual health is the affirmation of life in relationship with God, self, the community and the environment that celebrates wholeness” (Moberg, 2002, p. 48).

Drawing on the theoretical model proposed by NICA (1975), Fisher (1998) conducted a study in Australia with 98 high school teachers and 23 experts. In interviews, subjects were presented with questions from several well-known instruments designed to measure spirituality, namely the Spiritual Well-Being Scale (Ellison, 1983), the Spiritual Orientation Inventory (Elkins, Hedstrom, Huges, Leaf, & Saunders, 1988), the Mental, Physical and Spiritual Well-Being Scale (Vella-Brodrick & Allen, 1995), the Spiritual Assessment Inventory (Hall & Edwards, 1996), the Perceived Wellness Survey (Adams, Bezner, & Steinhardt, 1997) and the JAREL Spiritual Well-Being Scale (Hungelmann, Kenkel-Rossi, Klassen, & Stollenwerk, 1996). Exploratory factor analysis of the responses revealed four main factors (personal, communal, environmental and transcendental), confirming NICA’s theoretical model (1975). These results allowed Fisher (1998) to conclude that spiritual well-being reflects the extent to which people perceive that they live in harmony with themselves and others, with the environment and with the transcendental.

Based on the definition of SH (Fisher, 1998, p. 191), Fisher (1999) developed the Spiritual Health and Life-Orientation Measure (SHALOM) to promote research into the relationship between spirituality and health. The SHALOM was based on the four factors Fisher had identified in the earlier exploratory analytic study, (a) personal—items that measure sense of identity, self-awareness, joy in life, inner peace and meaning in life; (b) communal—items that measure love for others, forgiveness of others, trust between individuals, respect for others and kindness toward others; (c) environmental—items that measure the connection with nature, awe at breathtaking views, oneness with nature, harmony with the environment and a sense of wonder at the environment and (d) transcendental—items that measure personal relationship with the divine/God, worship of the Creator, oneness with God, peace with God and prayer in life. The instrument measures what responds consider an ideal state of SH (ideal component), as well as their actual experience of spiritual well-being (lived experience component).

The lived experience component of the SHALOM, also known as the Spiritual Well-Being Questionnaire (SWBQ; Gomez & Fisher, 2003), has been tested in several cultures (Fisher, 2010), such as Australia (Gomez & Fisher, 2003), Canada (Holder, Coleman, & Wallace, 2010), Germany (Rowold, 2011) and Portugal (Gouveia, Pais-Ribeiro, & Marques, 2008; Gouveia, Marques, & Ribeiro, 2009; Gouveia & Marques, 2012). In these studies, the SWBQ demonstrated good reliability and good predictive, discriminant and construct validity. SHALOM has also been validated through confirmatory factor analysis (CFA) in some of these countries (Fisher, 2010). A recent review of the ten questionnaires that measure spirituality as a universal experience concluded that the SWBQ was the only instrument that was valid, reliable and suitable for use in clinical contexts (Meezenbroek, Garssen, van den Berg, van Dierendonck, Visser, & Schaufeli, 2012).

Gomez and Fisher (2003) used CFA to assess the factorial validity of the SBWQ in two studies. In both studies, they tested four distinct models: a four-factor oblique model (assumes that factors are correlated with each other), a four-factor orthogonal model (assumes that the extracted factors are independent), a one-factor model (one general factor underlying 20 items) and a second-order hierarchical model (all four first-order orthogonal factors load on a single second-order factor, named spiritual well-being). In the first study, the fit indices of both the one-factor and four-factor orthogonal models fell outside the range considered to indicate good fit, but the all the fit indices calculated indicated that the second-order hierarchical model was a good fit to the data. In the second CFA study, both the four-factor oblique model and the second-order model showed satisfactory fit, but the one-factor and four-factor orthogonal models did not.

Fisher (2013) also presented CFA of three samples (*N*_1_ = 378, *N*_2_ = 460 and *N*_3_ = 409) testing two versions of the instrument, the original version (referred to as Personal Transcendent), which includes the words ‘God’, ‘Divine’ and ‘Creator’ in the items that measure the transcendental dimension, and another version in which these words were replaced by Transcendent, to remove the personification of the idea that takes us to a major, superior force (Deist bias). The model tested was a four-factor oblique with five items loading on each factor. In the first and second studies, in which the original SWBQ was used, this model provided an acceptable fit (*χ*^*2*^/*df* = 2.70; AIC = 574; ECVI = 1.51; IFI = .944; NFI = .914; TLI = .935; PNFI = .79; RMSEA = .067; CFI = .944 and *χ*^*2*^/*df* = 2.70; AIC = 575; ECVI = 1.25; IFI = .941; NFI = .909; TLI = .931; PNFI = .79; RMSEA = .061; CFI = .940, respectively). In the third study, Fisher divided the sample into two groups, one group (*n* = 231) completed the original SWBQ and the other group completed the modified SWBQ. In the modified version, the term used to refer to a divine entity (Allah, Angels, Buddha, Gaia, God, Universal Spirit etc.) was tailored to the particular beliefs of each respondent individual. The following fit indices were obtained: group 1: *χ*^*2*^/*df* = 2.97; AIC = 620; ECVI = 2.70; IFI = .899; NFI = .855; TLI = .882; PNFI = 0.74; RMSEA = .093; CFI = .898 and group 2: *χ*^*2*^/*df* = 1.66; AIC = 404; ECVI = 2.93; IFI = .940; NFI = .882; TLI = .930; PNFI = 0.74; RMSEA = .069; CFI = .939. Based on these results, Fisher concluded that the modified SHALOM can be used as a generic measure of spiritual well-being, in people with a wide variety of world views and cultures.

The factorial validity of the SWBQ also was tested in Germany by Rowold (2011) and in Portugal by Gouveia et al. (Gouveia et al., 2009, Gouveia & Marques, 2012). Rowold (2011) investigated the factorial validity of a German version of SWBQ, named SWBQ-G, and started by testing whether the four-factor model of spiritual well-being described by Gomez and Fisher (2003) could be replicated in a German sample. He concluded that the four-factor oblique model (target model) fitted the data significantly better than either a baseline (i.e. zero-factor) or a one-factor model. He then tested whether the four-factor model of spiritual well-being fitted the data significantly better than all plausible three-factor models (for example, if the relationship between personal and communal spiritual dimensions was set to 1). Again, the four-factor model proved a better fit to the data than any of the three-factor models tested. Finally, Rowold tested whether the second-order model fitted the data better than the four-factor oblique model, once again confirming the superior fit of the four-factor single-order model.

Gouveia et al. (2009) also tested a four-factor oblique model and found acceptable fit indices, although the wording of items 6 (worship of the Creator, translated to *admiração e respeito pelo Criador*), 8 (trust between individuals translated to *confiança entre as pessoas*) and 9 (self-awareness translated to *autoconhecimento/autoconsciência*) appeared problematic. Later Gouveia et al. (2012) confirmed that a four-factor oblique model and a hierarchical model both provided an acceptable fit to the Portuguese version of the Spiritual Well-Being Questionnaire (SWBQ_p_) although they reported that to ensure the quality of the instrument items 8, 9 and 15 (prayer life translated to *uma vida de meditação e/ou oração*) need to be eliminated or re-worded.

The gender invariance of the four-factor oblique model was confirmed in a sample of 3010 women and 1361 men using multi-group CFA (Gomez & Fisher, 2005b). In addition, an item response theory (IRT)-based analysis of data from 4462 respondents suggested that the instrument had good psychometric properties (Gomez & Fisher, 2005a).

Although a European-Portuguese version of the SWBQ has been validated in Portugal (Gouveia et al., 2009), we are not aware of any attempts to adapt and validate the SWBQ for the Brazilian population. A specifically Brazilian version is needed as there are significant linguistic and cultural differences between Brazil and Portugal. Therefore, the aim of this study was to fill this gap by (i) translating and culturally adapting the SWBQ for use in Brazil and (ii) analysing the psychometric properties (reliability, convergent, discriminant and factorial validity) of the Brazilian SWBQ (SWBQ_b_). To do this, we used a cross-validation approach (Byrne, 2016), identifying the best-fitting measurement model of this scale in a calibration sample (study 1), then confirming the results in a separate validation sample (study 2).

## Study 1

### Method

#### Participants

We recruited a non-probabilistic, intentional sample of 436 adults (> 18 years). The sample size was chosen to comply with the recommendation (Tabachnick & Fidell, 2001) that there should be at least ten respondents per item in validation studies.

The sample comprised 159 men and 277 women. The age of participants ranged from 18 to 79 years old (mean = 32.20 years; SD = 11.46). Most of them (56%) were *pardo* (an ethnicity/colour category used by the Brazilian Institute of Geography and Statistics in Brazilian censuses. It is a Portuguese word that encompasses various shades of brown, but is usually translated as ‘greyish-brown’). The rest were black (*n* = 99, 22.7%), white (*n =* 88, 19.7%), East Asian (*n =* 6, 1.4%) or Amerindian (*n =* 1, .2%). Over half of the participants (59.6%) were single and 33.5% were married. The majority (65%) had at least some secondary education. About half the participants (50.2%) were in paid work and 28.9% were students. The majority of participants (64.2%) reported a monthly family income of about 1.5 to 5 times the Brazilian minimum wage (R$ 937.00 or US$287.89). The distribution of declared religious belief was as follows: Catholic 36.2%, evangelical Christian 31%, Kardecist Spiritism 16.5%, other religion, not specified 10.3%, agnostic 12.5%, Candomble (Afro-American religion) 1.6%, atheist 1.4%, Buddhist .2% and Jewish .2%.

#### Instruments

Participants completed a ten-item sociodemographic questionnaire and the SHALOM.

The SHALOM was developed by Fisher (1999, 2010) and consists of 20 items, distributed over four dimensions: personal (items 5, 9, 14, 16 and 18), communal (items 1, 3, 8, 17 and 19), environmental (items 4, 7, 10, 12 and 20) and transcendental (items 2, 6, 11, 13 and 15). For each items, participants were asked to consider: “How important you think each area is for an ideal state of spiritual health?” (ideal component) and “How you feel each item reflects your personal experience most of the time?” (lived experience component, also known as the SWBQ). Responses were given using a five-point Likert type ranging from 1 = very low to 5 = very high. Following the suggestion of Fisher (2013) and Gouveia et al. (2009), a phrase was added at the beginning of the instrument: *If it seems more appropriate to you, please replace the word ‘God’ with ‘Transcendental’, ‘Cosmic Force’, ‘Universe’ or another similar expression which makes the item more meaningful to you.* Because of space constraints, only the SWBQ_b_ (lived experience component) results are reported here.

#### Procedures

After the study was approved by the Ethics Committee of Santa Cruz State University (reference no. 1325494), the research team approached potential participants individually or collectively, at times previously agreed with participating institutions to explain the goals of the research and invite them to participate. Those who expressed interest in participating were invited to read and sign a consent form. The instruments were administered both individually and collectively in the places where subjects were recruited (churches, spiritual centres, classrooms etc). The total time required to complete the instruments was about 20 min. Data were collected from students of five religious institutions with different faith traditions and from students from a public university. All questionnaires were checked for incomplete responses, and no missing values were detected.

#### Translation and cultural adaptation

We chose to work with the original English version of the SWBQ to avoid being influenced by any subtle changes of meaning introduced in the Portuguese translation of the instrument. Our cultural adaptation was based on the procedures recommended by Vallerand (1989) and Brislin (1970) for the cultural adaptation of psychological instruments and was carried out with the approval of the author of the original instrument. The steps were as follows: (1) Preparation of a preliminary version through translation and back-translation technique, using two translators and two back translators, two had a PhD in English language, two had a PhD in Psychology and had expertise in research into spiritual. (2) evaluation of the preliminary version and preparation of the experimental version, in order to check the accuracy of the preliminary translation. The back-translated version of the instrument was evaluated by a panel composed of four university professors (two translators and two researchers). Some of the technical terms used in the Brazilian version were revised to make them more compatible with the original and so that there was a consensus between the experts on the accuracy of the translated version. This step was also used to assess the clarity, comprehensibility and representativeness of the items (Almeida & Freire, 2003). (3) Content validity was verified by a committee consisting of three judges: two with PhDs in Psychology and one with expertise in spiritual measures. First the committee members rated how representative the items were of the latent construct individually, using a ten-point Likert scale ranging from 1 = not at all relevant or representative to 10 = extremely relevant or representative. From their responses, we calculated content validity index (CVI) scores (Waltz, Strickland, & Lenz, 1991), obtaining average values higher than 80% for all items, which provides support for the theoretical-conceptual adequacy of the items as measures of their respective target factors. (4) Pre-test of the translated version: a sample of 50 participants from the target population completed the preliminary version of the instrument (Hill & Hill, 2005), marking on the questionnaire any words or expressions that they did not understand. They were also to comment on the content of the instrument. (5) Final review—the orthography, grammar and punctuation of the final version were checked.

#### Statistical analyses

We calculated descriptive statistics (means, standard deviations, minima and maxima) and assessed the distribution of variables. Values of skewness and kurtosis between − 2 and + 2 were considered acceptable. These analyses were performed using IBM SPSS Statistics 20.

CFA using the estimation method maximum likelihood method (implemented in AMOS 6.0) was carried out to test the factorial validity of the original model (Gomez & Fisher, 2003) and other models used in validation studies in the literature. We used a minimum number of ten observations per item (Ding, Velicer, & Harlow, 1995). The fit of models was evaluated using a number of indices. Non-significant (*p* > .05) values of *χ*^2^ indicate acceptable fit, but this statistic is sensitive to sample size, i.e. in larger samples, the value tends to be significant. Jöreskog and Sörbom (1989) suggested using *χ*^2^/*df* to address this problem, and Ullman (2001) proposed *χ*^2^/*df* < 2.0 as the criterion of acceptable fit. We also calculated the following indices of fit: (a) comparative fit index (CFI) and goodness of fit index (GFI)—values range from 0 to 1 and values larger than .90 indicate adequate fit (Bentler & Bonett, 1980) although more recently, Hu and Bentler (1999) suggested > .95 as the criterion for good fit; (b) root mean square error of approximation (RMSEA)—values lower than .08 indicate adequate fit (Browne & Cudek, 1993), although more recently, Hu and Bentler (1999) suggested < .06 as the criterion for good fit.

#### Model description

Eight SWBQ_b_ measurement models identified in the literature were specified and tested (Fisher, 2013; Gomez & Fisher, 2003; Gouveia et al., 2009, Gouveia & Marques, 2012). Model 1 was a 20-item one-factor model. Model 2 was a hierarchical second-order model (four first-order factors; five items loading on each factor). Model 3 was a four-factor orthogonal model (four independent factors; 20 items). Model 4 was a four-factor oblique model (four correlated factors; 20 items). These four models were tested in the original validation of the instrument in an Australian sample (Gomez & Fisher, 2003). Model 5 was derived from a validation study of the Portuguese version of the SWBQ (Gouveia et al., 2009) in which the authors found inconsistencies and suggested rewording items 6, 8 and 9 to improve psychometric properties of the questionnaire. We deleted these three items, resulting in a new four-factor oblique model (personal factor, five items; communal, environmental and transcendental factors, four items each). Model 6 was the same as model 5, but with three correlations between item errors (1–2, 4–5 and 17–19). Model 7 was the same as the original four-factor oblique model, but without items 8, 9 and 15, as suggested in another Portuguese validation study (Gouveia & Marques, 2012), i.e. four-item personal, communal and transcendental factors and a five-item environmental factor. Finally, model 8 shared the factorial structure of model 7, but within-factor item error terms were correlated. Models 6 to 8 were re-specifications of the previous models based on post hoc analyses provided by the AMOS software, after considering the theoretical and substantive meaning of these modifications (Byrne, 2016).

### Results

#### Descriptive analysis

The mean item scores ranged from 3.31 ± 1.05 (item 8) to 4.32 ± 1.05 (item 6). The univariate normality values mostly fell within an interval associated with normal distribution. In only one case (item 6) did the kurtosis value fall outside the acceptable range (− 2 to + 2). The means of the SWBQ_b_ subscales scores were as follows: personal, mean = 3.99; SD = .71; communal, mean = 3.88; SD = .62; environmental: mean = 3.69; SD = .82 and transcendental, mean = 4.00; SD = .89.

#### Confirmatory factor analysis

First, the fit of the various factors of the scale (personal, communal, environmental and transcendental) was tested separately; these results are presented in Table 1.

**Table 1:** Absolute goodness-of-fit indices for the SWBQ_b_ factors in the calibration sample (*N* = 436)

	*X*^2^/*df*	GFI	CFI	RMSEA	AIC
Personal	3.26	.986	.982	.072	87.10
Communal	16.31	.924	.850	.188	152.33
Environmental	1.49	.993	.997	.034	27.45
Transcendental	5.12	.997	.982	.097	45.64

Table 1 shows that, generally, the SWBQ_b_ factors were a good fit to the data when analysed separately. However, the RMSEA values for the communal and transcendental factors were above the cutoff point (.08), although they were acceptable for the environmental and personal factors. The communal factor showed the worst fit, and the environmental factor showed an excellent fit to the data.

Table 2 presents the goodness-of-fit indices for each of the eight specified models.

**Table 2:** Absolute goodness-of-fit indices for eight CFA models of the SWBQ_b_ in the Brazilian calibration sample (*N* = 436)

	*X*^2^/*df*	GFI	CFI	RMSEA (90%IC)	AIC
Model 1—one-factor model	9.563	.663	.650	.140 (.134–.147)	1908.802
Model 2—hierarchical model	4.13	.858	.877	.085 (.078–.092)	998.07
Model 3—original orthogonal model	7.85	.749	.725	.126 (.119–.132)	1818.71
Model 4—original oblique model	4.17	.859	.877	.085 (.079–.092)	1009.418
Model 5—modified oblique model (without items 6, 8 and 9)	4.227	.881	.838	.086 (.078–.094)	760.723
Model 6—modified oblique model (without items 6, 8 and 9; correlation between error for items 1–2, 4–5 and 17–19).	3.370	.911	.924	.074 (.066–.082)	678.769
Model 7—modified oblique model (without items 8, 9 and 15)	4.249	.881	.897	.086 (.079–.094)	763.271
Model 8—modified oblique model (without items 8, 9 and 15; correlation between error for items 1–2, 4–5 and 17–19)	3.398	.908	.926	.074 (.066–.083)	678.064

Table 2 shows that the one-factor model (model 1) had unacceptable fit in terms of *χ*^2^/*df*, confirming the multidimensionality of the data. Next, we tested three four-factor models: the original hierarchical model (Gomez & Fisher, 2003) and the orthogonal model and oblique models proposed by Fisher (2013). The four-factor oblique model proved a better fit to the data than the one-factor model and the other three four-factor models, but the fit indices still did not meet the criteria for acceptable model fit. Next model 5 (without items 6, 8 and 9, which did not load significantly on their respective target factors) was tested, in line with a previous validation study conducted in Portugal (Gouveia et al., 2009). Participants’ responses to the three excluded items were highly skewed, indicating that these items did not provide good discrimination. The means and SDs for these items were as follows: item 6—mean *=* 4.32, SD = 1.05; item 8—mean *=* 3.31, SD = 1.05 and item 9—mean *=* 3.83, SD *=* .89. The fit indices for model 5 were substantially better, but still marginally lower than the recommended thresholds. We then carried out post hoc re-specification of the best fitting factor structures, adding three error covariance terms (items 1–2, 4–5 and 17–19), based on modification indices and semantic similarity between items, to yield model 6. As Table 2 shows that the values of the fit indices were acceptable for model 6. We then tested model 7, which was derived from a second Portuguese validation study (Gouveia & Marques, 2012) which also eliminated the items which did not load significantly on their target factor (items 8, 9 and 15), but was not as good a fit to the data as model 6. Finally, based on modification indices, we added three error covariance terms (items 1–2, 4–5 and 17–19) to yield model 8, which presented acceptable fit indices. In summary, both models 6 and 8 provided an acceptable fit to the data; however, model 8 had a lower AIC, and the AIC is an index of parsimony, so this suggests that it is the simplest model which offers a good fit to the data analysed.

## Study 2

### Method

#### Participants

The sample for study 2 consisted of 388 people (253 women and 135 men) with age ranging from 18 to 59 years old (mean = 30.59 years; SD = 9.44). The main ethnic groups represented were as follows: *pardo* 58%, black 23.7% and white 16.8%. Most of the subjects were single (62.9%), 31.7% were married and 4.6% were divorced. The majority of participants (54.4%) had at least some higher education, and 29.9% had at least some secondary education. The majority of the sample (51.8%) was employed, and 29.9% were students. With respect to family income, 43.3% received up to three times the Brazilian minimum wages, and 26.5% from three to five times the Brazilian minimum wages. Finally, the main religious affiliations declared by the participants were Catholic (36.6%), evangelical Christian (33.8%) and Kardecist Spiritism (12.6%). This sample was independent of the sample for study 1, although both samples were recruited in the same geographical area, a medium-sized city located in the northeast of Brazil.

#### Instruments

In study 2, we administered the two instruments used in study 1 and the Psychological Well-Being Scale (PWBS).

The 36-item Brazilian version of the Psychological Well-Being Scale (PWBS_b_), adapted from the Ryff and Essex (1992) version, was validated by Machado, Bandeira, and Pawlowski (2013) for the Brazilian population. This scale has six items in each dimension: positive relations with others (items 1, 7, 13, 19, 25 and 31), autonomy (items 2, 8, 14, 20, 26 and 32), environment mastery (items 3, 9, 15, 21, 27 and 33), personal growth (items 4, 10, 16, 22, 28 and 34), purpose in life (items 5, 11, 17, 23, 29 and 35) and self-acceptance (items 6, 12, 18, 24, 30 and 36). Responses are given using a six-point Likert scale with ‘totally disagree’ and ‘totally agree’ as the poles. The PWBS is based on a multidimensional model of psychological well-being; the dimensions are as follows: (a) Positive relations with others, characterised by warming, satisfying, trusting and satisfactory relationships, concern for others’ well-being, empathy, affection, intimacy and understanding of relationships. (b) Autonomy—consists of auto-determination and independence and encompasses capacity to think and act independently and resist social pressure. It involves internally motivated behavioural regulation and self-evaluation according to personal standards. (c) Environment mastery, defined as competence in managing the environment, includes complex adjustments of external activities and the creation and exploitation of opportunities, the capacity to choose and create contexts to satisfy one’s personal needs and values. (d) Personal growth—related to the perception that one continues to develop and grow as a person; also encompasses openness to new experiences and the perception that one is realising one’s potential. Finally, it involves the perception that one is improving over time and changing in ways that reflect an increase in self-knowledge and effectiveness. (e) Purpose in life, which is related to goals in life and a sense of directedness, represents the feeling there is meaning to one’s present and past life; encompasses the holding of beliefs that give life purpose, i.e. having aims and objectives for living. (f) Self-acceptance, characterised by a positive attitude to oneself, knowledge and acceptance of one’s multiple aspects, including one’s good and bad qualities, and positive feeling about one’s past life. In Brazil, Machado et al. (2013) reported the following indices for the six-factor oblique model of the scale: *χ*^*2*^*/df* = 1368.25/579; CFI = .95, NFI = .95, RMSEA (90% CI) = .066 (.061–.071) and CAIC = 2537.80.

#### Procedures and statistical analyses

The procedures and statistical analyses were identical to those used in study 1, with three exceptions. Reliability was determined by computing Cronbach’s alpha and the composite reliability statistics. Convergent validity was analysed by comparing the average variance extracted (AVE) for each factor with the factor’s correlation with other constructs and discriminant validity was evaluated by comparing the average variance extracted square root (√AVE) and the square of the correlation between the factors. All questionnaires were checked for incomplete responses, and no missing values were detected.

### Results

#### Confirmatory factor analysis

To verify that the best-fit model identified in study 1 (i.e. model 8) was generally applicable, we carried out cross-validation analysis in an independent (validation) sample. Preliminary analysis confirmed that no extreme values (outliers) were found and that the values for asymmetry and kurtosis were between − 2 and + 2. In addition, no post hoc modifications (from AMOS) were indicated.

**Fig. 1:**
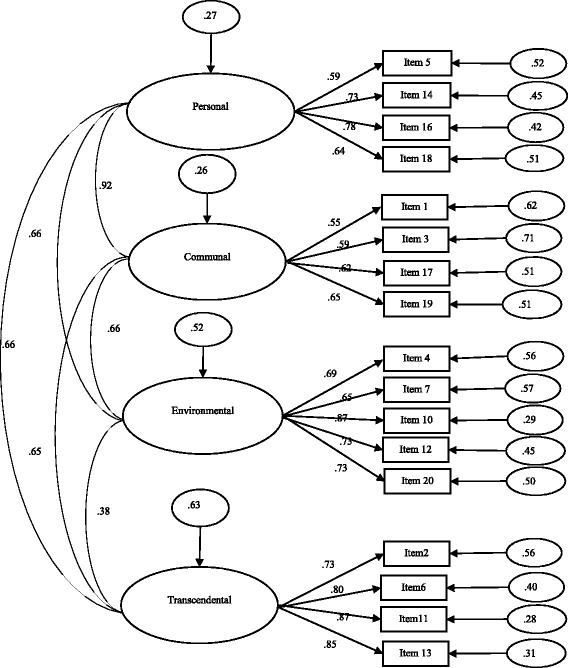
Measurement model with 17 items and four factors, confirmed in the validation sample. All saturation values are significant (*p* < .05)

The 17-item solution—a model without items 8, 9 and 15 and with correlation between error terms for some pairs of items 1–2 (*r* = .25), 4–5 (*r* = .31) and 17–19 (*r* = .40)—showed an acceptable fit to data from the validation sample (*χ*^*2*^*/df* = 3.030; GFI *=* .909; CFI *=* .931, RMSEA (90% CI) *=* .072 (0.064–.081); AIC = 632.637), thus confirming its structure (Fig. 1). All items had factor loadings greater than .50 for their respective target factors.

#### Reliability

Table 3 summarises the reliability results for the four dimensions of the original 20-item and for the shortened four-factor model (without items 8, 9 and 15).

**Table 3:** Cronbach’s alpha and composite reliability coefficients for the original and shortened versions of the SWBQ_b_

	Original version Cronbach’s alpha	Shortened version Cronbach’s alpha	Shortened version composite reliability
	SWBQ_b_	SWBQ_b-red_	SWBQ_b-red_
Communal	.74	.72	.71
Personal	.81	.78	.80
Environmental	.85	.86	.85
Transcendental	.88	.88	.87

All factors had values of .7 or higher, which is considered good (Malhotra, 2011).

#### Convergent and discriminant validity

Convergent and discriminant validity were investigated using the model tested and validated in this study. The convergent validity of the SWBQ_b_ was assessed in two ways: (1) we examined whether scores on the four SWBQ_b_ factors were correlated with scores on an independent measure, and (2) we evaluated the AVE values using Fornell and Larcker’s (1981) criteria (AVE ≥ .50 indicates acceptable convergent validity).

Table 4 summarises the results of the correlation analysis.

**Table 4:** Pearson’s correlations between SWBQ_b_ and PWBS dimensions

	PWBS					
SWBQ	PR	AU	EM	PG	PL	SA
Communal	.160**	.089	.154**	.145**	.154**	.156**
Personal	.134**	.217**	.252**	.181**	.232**	.229**
Transcendental	.111*	.096	.167**	.093	.142**	.104*
Environmental	.079	.150**	.181**	.132**	.132**	.138**

*PR* positive relations, *AU* autonomy, *EM* environmental mastery, *PG* personal growth, *PL* purpose in life, *SA* self-acceptance

*p < .05; **p < .01

As expected, there were positive correlations between SWBQ factors and PWBS factors. The highest correlations were between SWBQ-personal (related to self-awareness, inner peace, for example) and PWBS-purpose in life (the belief that one’s life is purposeful and meaningful), PWBS-self-acceptance (a breadth of wellness that includes positive evaluations of oneself and one’s past) and PWBS-autonomy (sense of self-determination). There were also positive correlations between SWBQ-communal (forgiveness toward others, trust between individuals and respect for others) and all the PWBS factors except autonomy. The SWBQ factor that was most weakly correlated with the PWBS factors was SWBQ-transcendental (which captures the respondent’s perception of his or her peace with God or personal relationship with the divine, for example). Nevertheless, SWBQ-transcendental was positively correlated with four PWBS factors: environmental mastery (.167), purpose in life (.142), positive relations (.111) and, finally, self-acceptance (.104).

Table 5 presents the results of the convergent and divergent validity analysis.

**Table 5:** Average variance extracted, square root of AVE and matrix of correlations between factors

Dimension	AVE	1	2	3	4
1. Personal	.47	**.69**			
2. Communal	.36	.92	**.60**		
3. Environmental	.55	.58	.66	**.74**	
4. Transcendental	.66	.66	.65	.38	**.81**

The values shown in bold are the square root of AVE

Convergent validity was analysed in terms of AVE, using a cutoff point of .50 (Malhotra, 2011). As shown in Table 5, the AVE values for two of the four factors were below the recommended cutoff. Inspection of the AVE values for all the factors suggests that the communal factor lack convergent validity (.36), the personal factor shows almost acceptable convergent validity (.47) and the environmental and transcendental factors show acceptable convergent validity (.55 and .66, respectively).

In this sample, 9 of the 17 items included the measurement model had target factor loadings ≥ .70 (range .55–.87, all *p* < .05). The items belonging to the communal factor had the lowest factor loadings.

Discriminant validity was evaluated by comparing the values of the √AVE with the square of the correlation between the factors (Table 5). According to Malhotra (2011) and Fornell and Larcker (1981), an √AVE that is higher than the coefficient of the correlation between factors provides evidence of discriminant validity. Table 5 shows that this criterion was not met with respect to the discrimination between the personal and communal factors (square roots of AVE were .69 and .60, respectively; both these values are lower than the correlation between the factors, which was .92), but was met with respect to the personal and environmental factors (square roots of AVE were.69 and .74, respectively, correlation between the factors was .58), personal and transcendental factors (√AVE = .69 and .81, respectively; *r* = .66) and environmental and transcendental factors (√AVE = .74 and .81, respectively; *r* = .65). The validity of the discrimination between the communal and environmental factors was marginal (√AVE = .60 and.74, respectively; *r* = .66). In summary, it can be seen from Table 5 that all the constructs had √AVE greater than most of the interfactor correlations, providing some evidence of discriminant validity.

## Discussion

The aim of this study was to develop and validate—using CFA—a Brazilian adaptation of the lived experience component of the SHALOM, also called SWBQ_b_. Construct validity was verified by cross-validation of the dimensionality of the scale and by assessing its convergent and discriminant validity. The reliability of the instrument was established by calculating Cronbach’s alpha and composite reliability statistics.

The results for the first three tested models in study 1 (in the calibration sample) are in accord with the results of the original study (Gomez & Fisher, 2003) and later results reported by Fisher (2013). All three studies indicate that neither a 20-item, one-factor model, a 20-item second-order hierarchical model with four first-order factors nor a four-factor orthogonal model provide a good fit to SWBQ data. The best-fit model was the four-factor oblique model. Our results also corroborate analyses of German and Portuguese samples. Rowold (2011) concluded that a four-factor oblique model (target model) fitted the data significantly better than the baseline model, a one-factor model and all plausible three-factor models. Gouveia et al. (Gouveia et al., 2009, Gouveia & Marques, 2012) found good fit indices for the second-order factor, namely spiritual well-being, but the best indices were found in the four-factor oblique model (*χ*^2^/*df* = 2.803, *p* < .0001; CFI = .92; RMSEA = .06).

However, based on the analysis of the internal consistency of the items and regression weights in the confirmatory analysis, the authors of the first Portuguese validation study (Gouveia et al., 2009) suggested that revision of the wording of items 9 (deals with self-awareness, loads on the personal factor), 6 (deals with worship of the Creator and loads on the transcendental factor) and 8 (deals with trust between individuals and loads on the communal factor) was needed. They noted that these items had also proved problematic in studies of the original version (Gomez & Fisher, 2005a, 2005b) and concluded that future research with the Portuguese version should either exclude or revise these items in order to improve the psychometric properties of the instrument.

Based on these analyses of the Portuguese version, we opted to test two additional models, a four-factor oblique model without items 6, 8 and 9 (Model 5) and another similar model without these three problematic items but with error correlations between items 1–2, 4–5 and 17–19 (model 6). Of the first six models tested, model 6 offered the best fit, corroborating the results of the first Portuguese validation study (Gouveia et al., 2009).

Finally, Gouveia et al. (Gouveia & Marques, 2012) re-tested the four-factor oblique model in a sample of 342 practitioners of physical activities in the Oriental tradition (age range 15–71 years, 53% women) and suggested further refinements, namely elimination of items 8 (communal factor; development of trust in others), 9 (personal factor; development of self-awareness) and 15 (transcendental factor; development of a meditation and/or prayer life). The authors concluded that their revised structural model of the SWBQ_p_ provided an adequate fit to the data. Furthermore, the internal consistency and composite reliability were satisfactory for all four factors, confirming the results of previous validation studies (Gomez & Fisher, 2003, 2005a, 2005b).

Inspired by this recent Portuguese study, we specified and tested two additional models, models 7 and 8, in this study. The results showed that model 8 (four-factor oblique model, without items 8, 9 and 15 and with correlated error terms for items 1–2, 4–5 and 17–19) provided the best fit to the data. Consistent with the Portuguese validation studies, CFA of model 8 revealed that this model provided a good fit to the Brazilian data (CFI and GFI > .90; RMSEA < .08). In this model, the target factor loadings for all items were significant and ranged from .59 to .87, providing evidence for the construct validity of the SWBQ_b_. In other words, CFA of the Brazilian samples showed that spiritual well-being can be conceptualised in terms of four domains (personal, communal, environmental and transcendental), as Gomez and Fisher (2003) originally proposed, although it seems that refinement of the SWBQ is needed to improve its construct validity. It also seems that Brazilians and Portuguese share some aspects of spirituality and religion as well as a language. In fact, Christianity is the dominant religion in both countries. According to the 2011 Census, 81% of the population of Portugal is Catholic, whilst 2010 Brazilian census indicated that around 65% of Brazilians consider themselves Catholic. Although Brazil is a spiritually diverse society as a result of the mixing of the traditions of the Roman Catholic Church, the religious traditions of African slaves, indigenous beliefs and, more recently, a growth in evangelical Protestantism, Roman Catholic precepts continue to have a significant impact on Brazilian society and culture, as they do on Portuguese society. The content of the eliminated items—which relate to development of trust in others, development of self-awareness and development of a meditation and/or prayer life—relates to key themes of the centuries-long Buddhist tradition of meditation, but has less relevance to Catholic and evangelical Christians, who made up 70.4% of the validation sample. This may be one of the reasons why these items did not function well in Brazilian and Portuguese culture.

When we analysed the relationships between SWBQ_b_ and PWBS factors to assess the convergent validity of the SWBQ_b_, we found 20 positive correlations out of 24 relationships that were tested, although they were weak (< .40). Previous international studies have reported positive correlations between spiritual well-being and psychological well-being among English samples (Fisher, 2011; Rowold, 2011). A validation study of SWBQ_p_, conducted in Portugal (Mangia, 2015) using the same instruments as in this study found evidence for the convergent validity of the SWBQ_p_ and also obtained a very similar number of positive correlations (19 correlations). The correlations reported by Mangia (2015) were similar in magnitude to those we observed, although slightly higher. The pattern of correlations was very similar in the two studies: positive correlations between the SWBQ personal factor and all PWBS factors. Both studies also found positive correlations between SWBQ-communal and PWBS-environmental mastery, PWBS-personal development, PWBS-positive relationships, PWBS-purpose in life and PWBS-self-acceptance. SWBQ-transcendental was not correlated with PWBS-personal development in either study. We found no correlation between SWBQ_b_-transcendental and PWBS-autonomy, but Mangia (2015) did find a very weak correlation between these two factors in the Portuguese versions of the instruments. Finally, SWBQ_b_-environment was positively correlated with all the PWBS factors except positive relationships. These results provide evidence of the convergent validity of the SWBQ_b_ and corroborate studies that have observed correlations between spiritual well-being and psychological well-being in other countries (Fisher, 2011; Mangia, 2015; Rowold, 2011).

The factors that contributed most to the convergent validity of SWBQ_b_ were the personal and communal factors, which means that the greater one’s sense of identity, perception of joy and meaning in life, self-awareness and inner peace and the better one’s relationships with others (i.e. the more respectful one is of others, the more kindness, trust and forgiveness one shows toward them), the greater one’s perceived psychological well-being, i.e. the greater one’s sense of independence and self-determination (autonomy), ability to manage one’s life (environmental mastery) and openness to new experiences (personal growth) and the more positive ones relationships with others (positive relations with others), the more meaningful one’s life seems (purpose in life) and the more positive one’s attitude to oneself and one’s past life (self-acceptance).

Still, with respect to the convergent validity, it is important to highlight that all items had target factor loadings greater than .50. This result is consistent with the Portuguese validation study (Gouveia & Marques, 2012), in which the AVE of the SWBQ_p_ varied between .43 and .59. Nevertheless, only two of the four factors had AVE values greater than .50 in our study, namely the environmental and transcendental factors. In other words, the analyses provided insufficient evidence of the convergent validity of the personal and communal factors, as the variance shared between these dimensions and its indicators is smaller than the measurement error variance. Conversely, the AVE values for the other two factors (environmental and transcendental) indicated adequate convergent validity.

Turning to discriminant validity, the results suggest that most of the factors in the SWBQ_b_ share more variance with its specific indicators than with the other constructs (Hair et al., 2014). However, caution is needed when considering the discriminant validity of the Communal factor, as the √AVE values for this factor were poor, particularly with regard to its relationship with the personal factor. In addition, the personal and communal factors were extremely highly correlated (*r* = 0.92), suggesting similarity and redundancy. The overlap between these two factors has also been noted in a study carried out in Hong Kong (Yuen, 2015). As our study is the first to assess the discriminant validity of the SWBQ factors using AVE, further research is required to confirm the overlap between the personal and communal factors.

In the 17-item solution, the reliabilities of the subscales were above the usual cutoff point (> .70) and similar to those found in many other validation studies (Fisher, 1999, 2013; Gomez & Fisher, 2003; Gouveia et al., 2008, Gouveia et al., 2009, Gouveia & Marques, 2012). In this study, in addition to calculating Cronbach’s alpha, we also computed the composite reliability of the subscales. All the latent factors had acceptable composite reliability (≥ .70; Fornell & Larcker, 1981). These results confirm the findings of Gouveia et al. (Gouveia & Marques, 2012) and indicate that the retained items contribute to the reliable measurement of the constructs (Fernandes, Vasconcelos, & Fernandes, 2013; Fernandes, Nunes, & Fernandes, 2015). Therefore, our results demonstrate that most of the instrument’s dimensions showed reliability at both the item and construct level (Maroco & Garcia-Marques, 2006).

This study has some limitations: (i) the sample was a non-intentional, non-probabilistic type and therefore may not be representative of the general population of Brazil; (ii) the sample was recruited from a single region of Brazil, given the enormous cultural and religious diversity of the country future studies in other regions are necessary; (iii) the sample was not gender balanced, making it impossible to verify the metric invariance for this independent variable and (iv) this is the first published validation of the SWBQ in the Brazilian cultural context. It is important that further research validating the SWBQ_b_ is carried out, paying special attention to convergent and discriminant validity, as there was some inconsistency in our results in these areas.

## Conclusions

In conclusion, this study supports Fisher’s spiritual well-being model (Fisher, 1998; Gomez & Fisher, 2003) and contributes substantially to the validation of the SWBQ_b_, although we found that a shortened, 17-item version of the original SWBQ offers some advantages in the Brazilian context. Our results are completely consistent with the studies carried out to validate the SWBQ_p_. Both studies used a Portuguese-language version of the SWBQ, and both concluded that a model which excluded three problematic items offered the best fit to the data.

This study provides evidence for the reliability, construct validity, mean variance and convergent validity of the SWBQ_b_, as well as some evidence for the discriminant validity of the four factors of the SWBQ_b_. It suggests that SWBQ_b_ is suitable for assessing spiritual well-being in the Brazilian cultural context, in the fields of Health Psychology, Clinical Psychology and Nursing and thus contributing to the elucidation of the role of spiritual well-being in mental and physical health and people’s ability to cope with chronic disease.

## Abbreviations

√AVE: Average variance extracted square root
AIC: Akaike information criterion
AMOS: Analysis of moment structures
AVE: Average variance extracted
CAIC: Consistent Akaike Information Criterion
CFA: Confirmatory factor analysis
CFI: Comparative fit index
CVI: Content validity index
ECVI: Expected cross-validation index
GFI: Goodness-of-fit index
IBGE: Instituto Brasileiro de Geografia e Estatística
IBM SPSS: Statistical Package for the Social Sciences by International Business Machines
IFI: Incremental fit index
IRT: Item response theory
JAREL: Initials of the four authors (Joann A, Ruth, Eileen, and Loretta)
NFI: Normed Fit Index (Bentler-Bonnet)
NICA: National Interfaith Coalition On Aging
PNFI: Parsimonious normed fit index
PWBS: Psychological Well-Being Scale
RMSEA: Root mean square error of aproximation
SHALOM: Spiritual Health And Life-Orientation Measure
SWBQ: Spiritual Well-Being Questionnaire
SWBQ-G: German Version of the Spiritual Well-Being Questionnaire
TC: Term of consent
TLI: Tucker-Lewis index

## Authors’ information

S.A.N.N.

Research Group in Human Performance, GPED.

Integrated Program for Public Health Studies, PIESC.

Research Group in Processes of Subjectivation and Contemporaneity.

Federal University of the Southern Bahia, Bahia, Brazil.

H.M.F.

Research Centre in Sports Sciences, Health Sciences and Human Development, CIDESD.

Research in Education and Community Intervention, RECI, Portugal.

University of Trás-os-Montes and Alto Douro.

J.W.F.

Over the last 24 years, John W. Fisher has completed three doctorates related to spiritual health and well-being, PhD (University of Melbourne), EdD and PhD (University of Ballarat/Federation University Australia). In his active retirement, John participates in research and supervises graduate students in University of Melbourne (Honorary Senior Fellow), Federation University Australia (Adjunct Associate Professor), the Education University of Hong Kong (Honorary Academic Adviser) and Glyndwr University, Wales (Visiting Professor). John’s Spiritual Health And Life-Orientation Measure (SHALOM) has been sought for use in well over 700 studies in 31 languages.

M.G.F.

Research Group in Human Performance, GPED.

State University of Santa Cruz, Bahia, Brazil.
